# Multidisciplinary team management for prevention of pneumonia and long-term weight loss after esophagectomy: a single-center retrospective study

**DOI:** 10.1007/s10388-020-00721-0

**Published:** 2020-02-06

**Authors:** Sanshiro Kawata, Yoshihiro Hiramatsu, Yuka Shirai, Kouji Watanabe, Tetsuyuki Nagafusa, Tomohiro Matsumoto, Hirotoshi Kikuchi, Kinji Kamiya, Hiroya Takeuchi

**Affiliations:** 1 Department of Surgery, Hamamatsu University School of Medicine, 1-20-1 Handayama, Higashi-ku, Hamamatsu, 431-3192 Japan; 2 Department of Perioperative Functioning Care and Support, Hamamatsu University School of Medicine, Hamamatsu, Japan; 3 Department of Nutrition, Hamamatsu University School of Medicine, Hamamatsu, Japan; 4 Department of Rehabilitation, Hamamatsu University School of Medicine, Hamamatsu, Japan

**Keywords:** Esophagectomy, Pneumonia, Weight loss, Perioperative care, Medical care team

## Abstract

**Background:**

In April 2017, we launched the multidisciplinary Hamamatsu Perioperative Care Team (HOPE) for all surgical patients. We developed a reinforced intervention strategy, particularly for esophagectomy. We herein report the outcomes of the HOPE at 2 years after commencement.

**Methods:**

A total 125 patients underwent esophagectomy and gastric conduit reconstruction for esophageal or esophagogastric junction cancer between January 2014 and December 2018 at the Department of Surgery in Hamamatsu University School of Medicine. The patients were divided into the pre-HOPE group including 62 patients who underwent esophagectomy before the introduction of the HOPE and the HOPE group including 63 patients who underwent esophagectomy after the introduction of the HOPE. The outcomes of surgery were compared between the two groups.

**Results:**

There were no significant differences in the clinicopathological characteristics between the two groups. The incidence rates of atrial fibrillation and pneumonia were significantly lower in the HOPE group than in the pre-HOPE group (6% vs. 19%, *p *= 0.027 and 14% vs. 29%, *p *= 0.037, respectively). The estimated calorie doses at the time of discharge were approximately 750 and 1500 kcal/day in the pre-HOPE group and the HOPE group, respectively. The body weight loss was significantly less in the HOPE group than the pre-HOPE group at 1, 3, 6, and 12 months postoperatively than that before the surgery (*p *< 0.001).

**Conclusions:**

The introduction of the multidisciplinary HOPE was associated with a significant reduction in the incidence of postoperative pneumonia and significantly less weight loss.

## Introduction

Surgery plays a key role in the radical treatment of esophageal cancer. Recent advances in surgical techniques and perioperative management have dramatically improved the mortality rate; however, esophagectomy remains a highly invasive procedure and is associated with higher postoperative complication and mortality rates than surgery for other gastrointestinal cancers [1–3]. Postoperative complications such as anastomotic leakage, pneumonia, and surgical site infections result in extended hospitalization and impaired quality of life (QOL) [4]. Complications following surgery for esophageal cancer have been reported to contribute to not only short-term prognosis but also long-term prognosis [4–6].

Following esophagectomy, oral intake is not possible for several days after surgery, and difficulty with oral intake can continue over a longer time period due to anastomotic leakage and dysfunction in swallowing, which renders weight loss a major issue [7]. Postoperative weight loss is associated with reduced long-term QOL [8] and long-term prognosis [9]. Therefore, prevention of postoperative weight loss is an important goal.

In recent years, the enhanced recovery after surgery (ERAS) program has been shown to be effective for esophageal cancer [10]. Furthermore, creating a multidisciplinary team care and providing treatment in accordance with the ERAS program have been shown to contribute to the reduction in respiratory complications and length of hospital stay [11, 12].

In April 2017, we launched a multidisciplinary team care called the Hamamatsu Perioperative Care Team (HOPE) at our institution for all surgical patients with the aim to improve the safety of perioperative care, long-term prognosis, and long-term QOL. In the current study, we evaluated the short- and long-term surgical outcomes of the HOPE after 2 years following its commencement in patients undergoing esophagectomy and gastric conduit reconstruction for esophageal or esophagogastric junction cancer.

## Patients and methods

### Patients

A total of 125 patients underwent esophagectomy and gastric conduit reconstruction for esophageal or esophagogastric junction cancer between January 2014 and December 2018 at the Department of Surgery in Hamamatsu University School of Medicine. The patients were divided into the pre-HOPE group including 62 patients who underwent esophagectomy before the introduction of the HOPE and the HOPE group including 63 patients who underwent esophagectomy after the introduction of the HOPE.

### The HOPE protocol for esophagectomy

The HOPE comprised surgeons, nurses, rehabilitation physicians, physiotherapists, speech-language-hearing therapists, dieticians, and pharmacists who acted in collaboration with the nutritional support team, infection control team, and palliative care teams (Table 1).

**Table 1:** HOPE program

Dental screening and professional cleaning
Cessation of smoking and drinking
Measurement of physical fitness
Respiratory exercise using a device
Nutritional screening and support
Sufficient pain control
Early ambulation
Early enteral nutrition via jejunostomy tube
Swallowing evaluation

For patients who were scheduled to undergo surgery, a dental surgeon evaluated the oral hygiene of the patient and cleaned the mouth as soon as possible after the first visit in our outpatient clinic. We also instructed the patients to clean their mouths themselves and observed them regularly before and after surgery. The patients were instructed to abstain from smoking and consuming alcohol for at least 4 weeks before surgery. The rehabilitation physician and the physiotherapist measured the parameters of physical strength such as gait speed and grip strength, and conducted the cardiopulmonary exercise test to determine the exercise tolerability of the patients. The patients were directed to do more than 40 min of aerobic exercises such as walking, and perform resistance exercises such as sit-ups and squat every day, before surgery. Physiotherapists commenced respiratory rehabilitation using an incentive spirometer and provided guidance regarding postoperative expectoration methods. In the pre-HOPE group, preoperative rehabilitation and oral care were left to self-management after the first instruction; whereas, the medical staff actively encouraged patients to implement preoperative rehabilitation and oral care in the HOPE group. The dietician and the nutritional support team started intervention to all esophageal cancer patients as soon as possible after the first visit in the outpatient clinic and evaluated the patient’s dietary intake. We measured serum albumin, prealbumin, cholinesterase, total lymphocyte count and hemoglobin as nutritional assessment. The dietician measured body composition such as arm circumference, triceps skinfolds, calf circumference, and measured lean body mass using bioelectrical impedance analysis and diagnosed patients with skeletal muscle less than 7.0 kg/m^2^ for men and 5.7 kg/m^2^ for women as sarcopenia according to the Asian Working Group on Sarcopenia [13]. We performed intensive interventions, especially for patients with albumin < 3.5 or non-volitional weight loss or sarcopenia. Our goal was to improve these nutritional indicators such as serum albumin, body weight, and skeletal muscle mass as much as possible before surgery.

Following the surgery, early ambulation was encouraged, while providing care to relieve pain. Intravenous nutrition was administered approximately 600 kcal/day until the tenth–fourteenth days after surgery. The elemental diet was started primarily at 10 kcal/h from the day of surgery via jejunostomy tube. The tube-feeding dose was gradually increased to 1200 kcal/day. At the time of oral intake initiation, the rehabilitation physician and the speech-language-hearing therapists performed videofluoroscopic and videoendoscopic examinations of swallowing in all the patients to evaluate the swallowing function. The meals were started with a dysphagia diet and, later, changed to a liquid diet in accordance with the improvement in the swallowing function. After the start of oral intake, tube feeding was increased or decreased based on the energy of oral intake. Enteral nutrition through jejunostomy was continued in the patients, even after the hospital discharge until there were satisfactory results in oral intake. The estimated oral intake calorie dose at the time of discharge was approximately 750 kcal/day in the pre-HOPE group. In the HOPE group, the oral intake was equivalent to that of the pre-HOPE group, and 300 kcal/day was added as enteral nutrition, and approximately 400 kcal/day was added as oral nutrition supplementation. In the HOPE group, the total calories at the time of discharge were approximately 1500 kcal/day. The feeding jejunostomy tube was removed approximately 3 months after surgery in the HOPE group. The dietician and the rehabilitation staff intervened regularly after discharge for 1 year after the surgery.

Based on our previous report [14], we decided to perform a computed tomography (CT) examination on the seventh day after surgery routinely in the HOPE group for early detection of postoperative complications.

### Surgical procedure

All patients underwent right transthoracic esophagectomy with D2 lymphadenectomy according to the 11th edition of the Japanese Classification of Esophageal Cancer [15] and gastric tube reconstruction. Thoracotomy is performed in the left lateral decubitus position. Thoracoscopy is performed in the left lateral decubitus position or prone position. The subtotal gastric tube was prepared and brought up into the left side of the neck mainly through posterior mediastinal route. We have performed hand-sewn anastomosis at the neck. The method of creating the gastric tube and the anastomosis method were not changed in both the groups.

The thoracic, abdominal, and cervical portions of the surgical procedure were performed sequentially in the pre-HOPE group. In the HOPE group, cervical and abdominal portions of the surgical procedure were performed at the same time in the supine position, with the objective to reduce the operative time. In the pre-HOPE group, laparoscopy was mainly performed; however, laparotomy was also performed with the aim of shortening the operative time and safely creating the ideal gastric tube in the HOPE group. A feeding jejunostomy tube was placed in cases that were considered to be of high risk due to general conditions, or salvage surgery after definitive chemoradiotherapy in the pre-HOPE group; however, it was placed in all patients in the HOPE group. Three staff surgeons performed surgery in the pre-HOPE group, and one staff surgeon has been added in the HOPE group.

### Postoperative complications

Postoperative complications that occurred within the first 6 months after esophagectomy were evaluated according to the Clavien–Dindo classification by the attending physicians [16]. In addition, surgical complications, such as ≥ grade III anastomotic leakage and ≥ grade III superficial incisional surgical site infections, and internal complications, such as atrial fibrillation, ≥ grade II pneumonia, were evaluated. Recurrent laryngeal nerve palsy (RLNP) was also evaluated as an internal complication because it is at risk for pneumonia, which is defined as grade II according to the Clavien–Dindo classification. Postoperative pulmonary complications were evaluated using the definitions by the American Thoracic Society, the Centers for Disease Control and Prevention, and the Utrecht Pneumonia Scoring System [17]. We classified pneumonia according to whether it occurred before or after the start of the meal because the incidence of pneumonia after the start of the meal may have been caused by aspiration.

Anastomotic leakage was defined as the presence of signs indicating clinical leakage and/or findings of radiographic leakage by esophagogram or CT examination. RLNP was defined as the presence of laryngoscopic vocal cord palsy. Surgical site infections were diagnosed according to the definition by the Centers for Disease Control and Prevention.

### Assessment of postoperative body weight and skeletal muscle mass

The changes in body weight were examined at 1, 3, 6, and 12 months after the surgery by comparing with the preoperative body weight, which was defined as 100% for all patients. The evaluation of muscle mass was based on the psoas muscle index (PMI, cm^2^/m^2^), which was calculated as the sum of bilateral psoas muscle mass, determined by manual tracing at the third lumbar vertebral level using computed tomography (CT) images, divided by height squared [18]. The PMI was measured using the last CT images obtained before the surgery and the first CT images obtained between 4 and 6 months after the surgery. Patients with signs of cancer recurrence by 6 months after the operation were excluded from the nutritional and the skeletal muscle assessments.

### Statistical analysis

The features of distributions are presented as mean ± standard deviation (SD), or median and interquartile range (IQR) for variables with a skewed distribution, or frequency [proportion (%)]. Differences between groups in categorical variables were tested using Chi-square or Fisher’s exact test and for continuous data the Student *t* test or the Mann–Whitney *U* test were used. Two-way analysis of variance was used to compare body weight, serum albumin and PMI change. A *p* value of < 0.05 was considered to be statistically significant. Statistical analyses were performed with the IBM SPSS statistics 25.0 for Windows (IBM, NY, USA).

## Results

The clinical characteristics of the study cohort of 125 patients are shown in Table 2. There were no significant differences in age, sex, histological subtype, tumor location, cStage, preoperative treatment, preoperative body weight, preoperative PMI, and thoracic surgical approach between the two groups. The number of open abdominal surgeries has increased in the HOPE group with the aim of shortening the operative time and safely creating the ideal gastric tube than the pre-HOPE group (40% vs. 3%, *p *< 0.001). Because of the higher proportion of lower thoracic and abdominal esophageal cancer in the HOPE group, less patients in the HOPE group underwent cervical lymph node dissection than pre-HOPE group (73% vs. 90%, *p* = 0.011). The operative time was significantly shorter in the HOPE group (545 min) than the pre-HOPE group (716 min; *p *< 0.001). However, there was no significant difference in the estimated blood loss between the two groups.

**Table 2:** Patient characteristics

	Pre-HOPE *n* = 62	HOPE *n* = 63	*p* value
Age	65 (10)	68 (10)	0.112
Sex, male/female (%)	52 (84)/10 (16)	57 (90)/6 (10)	0.201
Histologic subtype (%)			0.260
Squamous cell carcinoma	56 (90)	51 (81)	
Adenocarcinoma	6 (10)	11 (17)	
Others	0 (0)	1 (2)	
Location of tumor (%)			0.338
Cervical esophagus	0 (0)	1 (2)	
Upper thoracic esophagus	5 (8)	8 (13)	
Mid-thoracic esophagus	36 (58)	26 (41)	
Lower thoracic esophagus	17 (27)	21 (33)	
Abdominal esophagus	4 (6)	7 (11)	
cStage (%)			0.185
0, I	30 (48)	20 (32)	
II	16 (26)	16 (25)	
III	14 (23)	24 (38)	
IV	2 (3)	3 (5)	
Preoperative treatment (%)			0.584
None	34 (55)	34 (54)	
Chemotherapy	25 (40)	23 (36)	
Chemoradiotherapy	3 (5)	6 (10)	
Preoperative body weight (kg)	58.2 ± 10.5	56.9 ± 78.8	0.463
Preoperative PMI (cm^2^/m^2^)	5.6 ± 2.0	5.0 ± 1.6	0.201
Preoperative white blood cell count (cells/µL)	5651 ± 1905	5336 ± 1955	0.363
Preoperative hemoglobin (g/dL)	12.6 ± 1.8	12.1 ± 1.7	0.154
Preoperative serum albumin (g/dL)	4.0 ± 0.3	4.0 ± 0.4	0.869
Thoracic approach (%)			0.522
Thoracotomy	17 (27)	18 (29)	
Thoracoscopy	45 (73)	45 (71)	
Abdominal approach (%)			< 0.001
Laparotomy	2 (3)	25 (40)	
Laparoscopy	60 (97)	38 (60)	
Cervical lymph node dissection (%)	56 (90)	46 (73)	0.011
Jejunostomy	10 (16)	63 (100)	< 0.001
Operative time (min)	716 (150)	545 (93)	< 0.001
Estimated blood loss (mL)	200 (301)	206 (273)	0.336

Bold values are presented as median (IQR) or mean ± SD

*PMI* psoas muscle index

The incidence of ≥ grade III anastomotic leakage was lower in the HOPE group than in the pre-HOPE group, albeit without statistical significance (5% vs. 15%, *p *= 0.060) (Table 3). The incidence rates of atrial fibrillation and pneumonia was significantly lower in the HOPE group than in the pre-HOPE group (6% vs. 19%, *p *= 0.027 and 14% vs. 29%, *p *= 0.037, respectively). Especially, the incidence rate of pneumonia after the start of meal was significantly lower in the HOPE group than in the pre-HOPE group (3% vs. 16%, *p *= 0.012). When comparing only cases with cervical lymph node dissection, the incidence of pneumonia after the start of the meal was significantly less in the HOPE group than in the pre-HOPE group [1/46 (2%) vs. 10/56 (18%), *p* = 0.010]. The incidence rate of ≥ grade II RLNP tended to be lower in the HOPE group than in the pre-HOPE group (6% vs. 16%, *p *=0.073). In the pre-HOPE group, there were 7 RLNP cases (11%) with pneumonia; however, there was no RLNP case with pneumonia in the HOPE group. There was no perioperative mortality in either group.

**Table 3:** Incidence of postoperative complications and mortality

	Pre-HOPE *n* = 62	HOPE *n* = 63	*p* value
Any complication			
≥ Grade II	42 (68%)	38 (60%)	0.249
≥ Grade III	15 (24%)	14 (22%)	0.480
Anastomotic leakage			
≥ Grade II	9 (15%)	7 (11%)	0.382
≥ Grade III	9 (15%)	3 (5%)	0.060
Superficial incisional surgical site infection			
≥ Grade III	1 (2%)	2 (3%)	0.506
Atrial fibrillation			
≥ Grade II	12 (19%)	4 (6%)	0.027
Pneumonia			
≥ Grade II	18 (29%)	9 (14%)	0.037
Pneumonia before the start of meal			
≥ Grade II	8 (13%)	7 (11%)	0.368
Pneumonia after the start of meal			
≥ Grade II	10 (16%)	2 (3%)	0.012
Recurrent laryngeal nerve palsy			
≥ Grade I	25 (40%)	19 (30%)	0.158
≥ Grade II	10 (16%)	4 (6%)	0.073
≥ Grade III	3 (5%)	4 (6%)	0.509
Mortality	0	0	–

There was no difference in the length of postoperative intensive care unit stay between the two groups (Table 4). The initiation of oral intake was significantly later in the HOPE group than the pre-HOPE group (11 vs. 8 days, *p *=0.003), and the postoperative hospital stay was significantly longer in the HOPE group than pre-HOPE group (31 vs. 23 days, *p *=0.010).

**Table 4:** Perioperative outcomes

	Pre-HOPE *n* = 62	HOPE *n* = 63	*p* value
Postoperative total ICU stay (days)	3 (1)	3 (1)	0.127
Postoperative hospital stay (days)	23 (24)	31 (17)	0.010
Start of oral intake (days)	8 (4)	11 (6)	0.003

Values are presented as median (IQR)

*ICU* intensive care unit

**Fig. 1:**
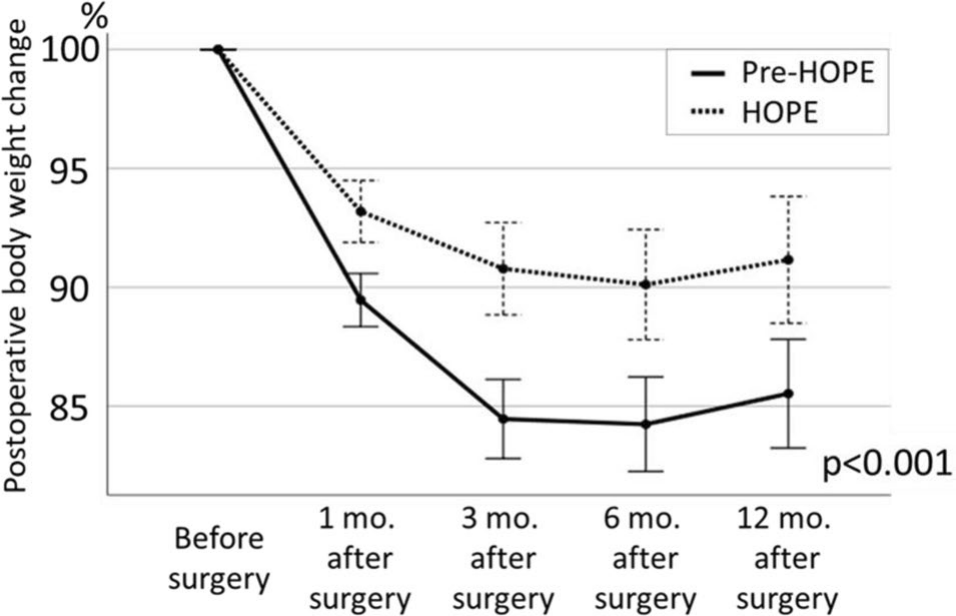
Postoperative body weight change in each group. The data are shown as mean and standard error

**Fig. 2:**
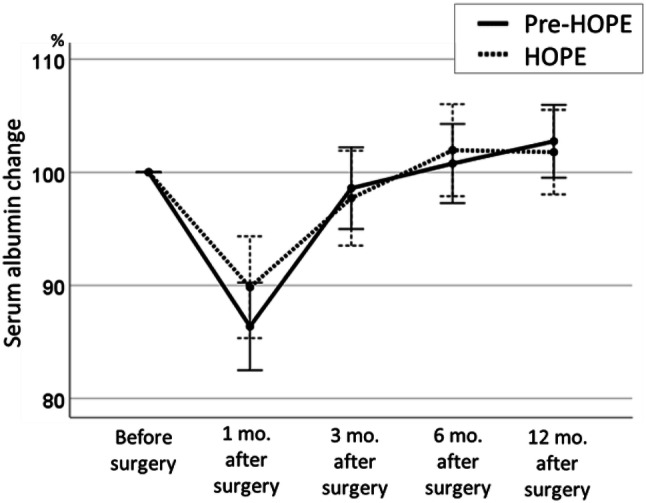
Serum albumin change in each group. The data are shown as mean and standard error

**Fig. 3:**
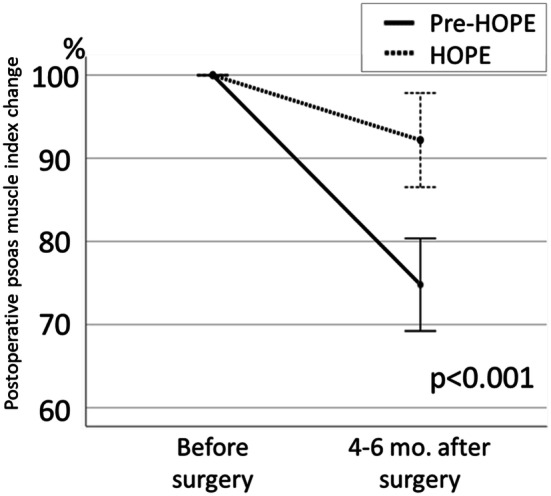
Psoas muscle index change in each group. The data are shown as mean and standard error

Based on the preoperative body weight set as 100%, the mean body weights at 1, 3, 6, and 12 months postoperatively were 93.6% ± 4.6%, 90.7% ± 6.1%, 90.3% ± 7.9%, and 91.2% ± 9.0%, respectively, in the HOPE group and 89.7% ± 4.2%, 84.6% ± 5.9%, 84.3% ± 6.7%, and 85.5% ± 7.9%, respectively, in the pre-HOPE group, indicating that the weight loss in the HOPE group was significantly less than that in the pre-HOPE group (*p *<0.001; Fig. 1). When comparing serum albumin changes at 1, 3, 6, and 12 months postoperatively, there was no significant difference between the two groups in two-way analysis of variance (*p* = 0.768; Fig. 2). Albumin change at 1 month after surgery tended to be lower in the HOPE group than pre-HOPE group, but there was no significant difference. Finally, the loss of postoperative PMI was also significantly lower in the HOPE group than the pre-HOPE group (92.2% ± 21.3% vs. 74.8% ± 21.9%, *p *<0.001; Fig. 3).

## Discussion

Esophagectomy is associated with higher rates of postoperative complications [1]. Respiratory complications are considered as contributing factors in the majority of postoperative deaths following esophagectomy [19]. Guidance for no smoking [20], oral care [21, 22], preoperative respiratory rehabilitation [23, 24], early postoperative ambulation [25], breathing exercise using an incentive spirometer [26], and adequate pain relief [27] are effective in reducing postoperative respiratory complications. The present study revealed that the implementation of the HOPE was associated with a significant reduction in the incidence of postoperative pneumonia, especially after the start of the meal. We believe that it is most important to work on bundle management to prevent pneumonia, not just one particular thing.

Dental screening and professional cleaning, cessation of smoking, respiratory exercises using a device, and early ambulation were performed in both the pre-HOPE and HOPE groups; whereas, the swallowing evaluation was performed only in the HOPE group. In the pre-HOPE group, preoperative rehabilitation and oral care were left to self-management after the first instruction; whereas, the medical staff actively encouraged patients to implement preoperative rehabilitation and oral care in the HOPE group. We believe that active coaching was important in preoperative management. Furthermore, the incidence of RLNP is higher and the fasting duration is longer following esophagectomy than other gastrointestinal surgeries; the elevation of the larynx, which can be impaired due to the invasiveness of the surgery, is also associated with a higher incidence of swallowing dysfunction in patients undergoing esophagectomy [28]. Therefore, after the introduction of the HOPE, as a rule, videoendoscopic and videofluoroscopic examination of the swallowing was performed at the time of oral intake initiation. In addition, the meals were provided in a form suited to each individual’s swallowing function. In the pre-HOPE group, there were 7 RLNP cases (11%) with pneumonia; however, there was no RLNP case with pneumonia in the HOPE group. We think that our protocol to prevent aspiration was effective, especially in patients with RLNP.

The incidence of pneumonia after the start of the meal may have been caused by aspiration. Laryngeal elevation is greatly affected by surgical procedures in the neck and that may cause swallowing dysfunction and aspiration. Therefore, when comparing only cases with cervical lymph node dissection, the incidence of pneumonia after the start of the meal was significantly less in the HOPE group than in the pre-HOPE group (2% vs. 18%, *p* = 0.010). We believe that HOPE management was important for the prevention of pneumonia after the start of the meal, not because of the surgical procedures difference in the neck.

Jejunostomy tube feeding is useful to prevent postoperative malnutrition. Dietitian-controlled proactive intervention was reported to reduce postoperative weight loss in patients undergoing surgery for esophageal cancer [29]. Following the implementation of the HOPE, all patients were evaluated and received guidance by the dietician prior to the surgery and nutritional support was provided if necessary. With jejunostomy, enteral feeding was commenced on the day of the surgery as a rule. Even in the patients with swallowing dysfunction, we believe that weight loss was consequently reduced by improving oral intake without aspiration based on the cooperation with the rehabilitation department and through the maintenance of tubal feeding via the jejunostomy in accordance with the patient’s swallowing function. At all points in time after the surgery, including 1, 3, 6, and 12 months, the weight loss was significantly less in the HOPE group than in the pre-HOPE group. Through the HOPE intervention, we were able to maintain a good nutritional state for the relatively long period of 1 year. Furthermore, maintaining body weight over the long term is expected to help maintain the long-term QOL and prolong long-term survival.

There was no significant difference in the incidence of ≥ grade II anastomotic leakage between the HOPE group and the pre-HOPE group (11% vs. 15%, *p* = 0.382). However, the incidence of ≥ grade III anastomotic leakage was remarkably lower in the HOPE group than the pre-HOPE group, albeit without statistical significance (5% vs. 15%, *p* = 0.060). We reported that CT is an objective and noninvasive screening method for the detection of complications after esophagectomy [14]. Hence, we decided to perform CT examination on the seventh day after surgery routinely in the HOPE group. As a result, the early diagnosis of anastomotic leakage could prevent the patients from developing serious complications.

Atrial fibrillation is the most common sustained cardiac arrhythmia in clinical practice. New-onset atrial fibrillation is not uncommon after noncardiac surgery, with reported incidence rates 3% [30]. The underlying causes and the triggers for atrial fibrillation include systemic inflammation, increased adrenergic tone, electrolyte abnormalities, anemia, hypothermia, hypoxia, and hypervolemia [31]. One potential reason for the reduced rate of atrial fibrillation in the HOPE group is the shortened operative time, but the preoperative and postoperative volume management in collaboration with the nutrition support team might have also led to the reduced rate of atrial fibrillation observed in the HOPE group. In our previous randomized control study that compared enteral and parenteral nutrition after esophagectomy, parenteral nutrition significantly related in higher urine output, more body weight loss, and volume management failure [32]. Therefore, we think that enteral nutrition had a better water balance and may reduce atrial fibrillation in the HOPE group. In general, pneumonia is known to cause atrial fibrillation; therefore, the decrease in the incidence of pneumonia may be one of the reasons why atrial fibrillation decreased in the HOPE group.

There were fewer cases than required to elucidate long-term hospitalization due to complications; however, the median length of hospital stay was significantly prolonged in the HOPE group. There are two potential reasons to account for this outcome: the timing of oral intake initiation and the delay in the increase of food intake. Time was needed to adjust the contents of the meals after discharge and to teach enteral nutrition to be implemented at home. The current study results contradict the findings of previous studies showing that the ERAS protocol led to shorter hospital stays [10]. In surgeries where relatively early patient recovery is expected, such as surgery for colon cancer, shortened hospital stays may be useful for early recovery of activities of daily life. However, in highly invasive surgeries such as esophagectomy, patients often remain on bed rest at home despite early discharge; therefore, continuation of active rehabilitation during hospitalization may be useful for long-term recovery of activities of daily life. A systematic review of the nutritional consequences of esophagectomy found that the weight changes were most significant in the first 6 months after surgery and that 27–95% of the patients failed to return to their preoperative levels [7], suggesting that the early weight loss sustained postoperatively was not reversed. Despite the longer hospital stay observed in the HOPE group, the patients received reliable nutrition therapy during the hospitalization and were prepared to be able to continue the therapy at home. Reducing the risk of malnutrition after discharge and controlling long-term weight loss are important goals. Recently, the Essential Strategy for Early Normalization after Surgery with Patient’s Excellent Satisfaction (ESSENSE) program was implemented to target patient-centered outcomes instead of efforts to reduce the hospitalization period [33]. The team medical care described in the current study is expected to improve the long-term QOL of patients.

The current study has several limitations. First, this was a retrospective analysis performed at a single institution. In addition, the groups were treated at different time periods, and the possibility exists that a learning curve in the surgical techniques and other confounders might have contributed to the outcomes.

## Conclusion

The introduction of the multidisciplinary HOPE was associated with a significant reduction in the incidence rate of postoperative pneumonia and significantly less weight loss in patients with esophageal or esophagogastric junction cancer who underwent esophagectomy and gastric conduit. Improving team proficiency is expected to reduce the complication rates overall and to improve long-term QOL and prognosis in these patients.
